# A novel small molecule inhibitor of MDM2-p53 (APG-115) enhances radiosensitivity of gastric adenocarcinoma

**DOI:** 10.1186/s13046-018-0765-8

**Published:** 2018-05-02

**Authors:** Hanjie Yi, Xianglei Yan, Qiuyun Luo, Luping Yuan, Baoxia Li, Wentao Pan, Lin Zhang, Haibo Chen, Jing Wang, Yubin Zhang, Yifan Zhai, Miao-Zhen Qiu, Da-Jun Yang

**Affiliations:** 1 Department of Experimental Research, State Key Laboratory of Oncology in South China, Collaborative Innovation Center for Cancer Medicine, Sun Yat-Sen University Cancer Center, 651 Dongfeng Road East, Guangzhou, 510060 China; 2 YinZhou hospital affiliated to medical school of NingBo University, NingBo, 315000 ZheJiang Province China; 3 Peking University shenzhen hospital, Shenzhen, 518063 China; 4 Guangzhou Red Cross Hospital, Guangzhou, 510060 China; 5 Ascentage Pharma, Taizhou, 225300 Jiangsu China; 6 Suzhou Ascentage Pharma Inc., Jiangsu, 215123 China; 7 Department of Medical Oncology, Sun Yat-Sen University Cancer Center; State Key Laboratory of Oncology in South China, Collaborative Innovation Center for Cancer Medicine, 651 Dongfeng Road East, Guangzhou, 510060 China

**Keywords:** Small molecule inhibitors, MDM2, p53, Gastric cancer, Radiation, Apoptosis

## Abstract

**Background:**

Gastric cancer is the leading cause of cancer related death worldwide. Radiation alone or combined with chemotherapy plays important role in locally advanced and metastatic gastric adenocarcinoma. MDM2–p53 interaction and downstream signaling affect cellular response to DNA damage which leads to cell cycle arrest and apoptosis. Therefore, restoring p53 function by inhibiting its interaction with MDM2 is a promising therapeutic strategy for cancer. APG-115 is a novel small molecule inhibitor which blocks the interaction of MDM2 and p53. In this study, we investigated that the radiosensitivity of APG-115 in gastric adenocarcinoma in vitro and in vivo.

**Methods:**

The role of APG-115 in six gastric cancer cells viability in vitro was determined by CCK-8 assay. The expression level of MDM2, p21, PUMA and BAX in AGS and MKN45 cell lines was measured via real-time PCR (RT-PCR). The function of treatment groups on cell cycle and cell apoptosis were detected through Flow Cytometry assay. Clonogenic assays were used to measure the radiosensitivity of APG-115 in p53 wild type gastric cancer cell lines. Western blot was conducted to detect the protein expressions of mdm2-p53 signal pathway. Xenograft models in nude mice were established to explore the radiosensitivity role of APG-115 in gastric cancer cells in vivo.

**Results:**

We found that radiosensitization by APG-115 occurred in p53 wild-type gastric cancer cells. Increasing apoptosis and cell cycle arrest was observed after administration of APG-115 and radiation. Radiosensitivity of APG-115 was mainly dependent on MDM2-p53 signal pathway. In vivo, APG-115 combined with radiation decreased xenograft tumor growth much more significantly than either single treatment. Moreover, the number of proliferating cells (Ki-67) significantly decreased in combination group compared with single treatment group.

**Conclusions:**

In summary, we found that combination of MDM2-p53 inhibitor (APG-115) and radiotherapy can enhance antitumor effect both in vitro and in vivo. This is the first report on radiosensitivity of APG-115 which shed light on clinical trial of the combination therapy of radiation with APG-115 in gastric adenocarcinoma.

**Electronic supplementary material:**

The online version of this article (10.1186/s13046-018-0765-8) contains supplementary material, which is available to authorized users.

## Background

Gastric cancer (GC) is the second most common cause of cancer-related death worldwide [1]. The incidence of GC varies significantly from one part of the world to another and it is particularly common in Eastern Asia, especially in China [2]. Although surgery is the main treatment of GC, chemo radiotherapy also plays an important role in neoadjuvant, adjuvant and palliative treatment of GC [3–7]. Radiosensitivity determines the treatment efficacy of ionizing radiation. p53 which is a tumor suppressor protein, known as ‘guardian of the genom’, plays a critical role in regulating stress responses such as DNA damage by ionizing radiation and also mediating a series of proteins participating in cell cycle, check-points, DNA repair and cell apoptosis. Because p53-dependent cell cycle arrest and apoptosis are detrimental to the growth and survival of normal cell, the activities of p53 are rigorously regulated in normal cells and tissues. Murine double minute 2 (MDM2) is the chief negative regulator of p53 [8]. MDM2 restrains p53 activity by binding and cutting off p53 from p53 target gene promoters [9, 10] Moreover, MDM2 contains a RING domain with E3 ubiquitin ligase activity, which can ubiquitinate p53 resulting in p53 degradation through a negative feedback loop [11, 12]. The important role of MDM2-regulated inhibition of p53 signaling pathway has been proved previously in vivo [13–15]. Therefore, MDM2 is critical for normal p53 signaling and cellular function. In response to DNA damage, p53 plays a vital role as a transcriptional factor that regulates a number of downstream targets. For example, p21 can induce cell cycle arrest [16, 17]. MDM2 negatively regulates the function of p53 retaining wild-type and increases response to ionizing radiation [18–20]. Several therapeutic strategies have been reported to activate p53 by disrupting the negative control by MDM2 [21]. Several studies have demonstrated the MDM2-p53 inhibitors can enhance the radiosensitivity in lung cancer, prostate cancer and colon cancer in vitro and in vivo [22–24]. APG-115 is a novel MDM2-p53 inhibitor, which have higher affinity to MDM2 than other MDM2-p53 inhibitors (Additional file 1). Because the important effect of both p53 and MDM2 in the regulation of ionizing radiation damage, the combination of APG-115 and radiation may provide a hopeful therapeutic remedy in gastric adenocarcinoma. In this study, we analyzed the capability of APG-115 to enhance radiation response in gastric cancer both in vitro and in vivo. Moreover, we preliminary explored the potential molecular mechanisms involving in the radiosensitivity of APG-115.

## Methods

### Reagent and antibodies

**Fig. 1:**
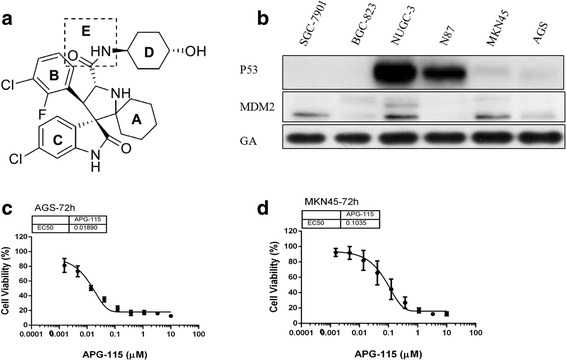
The Chemical structure of novel MDM2-p53 antagonists APG-115 and APG-115 inhibited p53 wild-type gastric cancer cells growth. (**a**) The structure of novel MDM2-p53 antagonist APG115. (**b**) p53 and MDM2 protein level in six untreated gastric cancer cell lines. (**c**, **d**) Cell proliferation was measured by CCK-8 after incubated for 72 h

APG115 was provided by Ascentage Pharma Group Inc. (Suzhou, China). The structure of the novel MDM2-p53 interaction antagonist APG-115 is shown in Fig. 1a. The compound was dissolved in dimethylsulfoxide (DMSO; Sigma Aldrich, MO, USA) at a stock concentration of 40 mM. The final concentration of DMSO in culture media did not exceed 0.1%. Antibodies against MDM2, p53, p21, PUMA, BAX, PAPR-1, γH2AX and Ki67 were purchased from Santa Biotechnology Inc. All other antibodies were obtained from Cell Signaling Technology. Cell culture media and supplements were purchased from Life Technologies, Inc.

### Cell line and cultures

All human gastric cancer cell lines (SGC-7901, BGC-823, NUGC-3, N87, AGS and MKN45) were purchased from Cobioer Biosciences Co.LTD (Nanjing, China). These cell lines were verified on the basis of cell morphology and genomic short tandem repeat (STR) profile of each cell line 4 to 6 months before starting study.Cells were incubated in RPMI 1640 medium with 10% fetal bovine serum (Gibico, CA, USA) and 1% penicillin-streptomycin (100 Units/ml penicillin and 100 μg/ml streptomycin) at 37 °C in a humidified atmosphere with 5% CO_2_.

### In vitro experiment treatment groups

Gastric cancer cells (AGS, MNK45) were treated with APG-115 (20 nmol/L, 200 nm/L) as APG-115 group, radiation as radiation group, both radiation and APG-115 (20 nmol/L, 200 nm/L) as combination group, and vehicle-only as control group for the appropriate time in each analysis. Cells of radiation and combination groups received radiation dose of 2, 6, or 10Gy in cell viability assay, and 4Gy in other analyses.

### In vitro and in vivo ionizing radiation

In vitro, the cells were irradiated with different doses and times at room temperature using X-ray in an X-ray irradiator RS2000 (RAD SOURCE, USA). In vivo, only the tumor was irradiated. The other parts of mice were shielded by lead.

### Cell viability assays

To evaluate the antitumor effect, cell proliferation of AGS (4 × 10^3^) and MKN45 (4 × 10^3^) cells were seeded in 96-well plates, and treated with each regimen for 72 h. Cell proliferation was measured with Cell Counting Kit-8 (CCK-8, Dojindo, Japan) following the manufacturer’s instruction. This experiment was done in triplicate.

### Quantification of mRNA expression

We uesd Premier 5.0 software to design the primers and compared it with BLAST in NCBI web [1, 25]. Relative mRNA levels of MDM2, p21,PUMA and BAX were quantified via real-time PCR (RT-qPCR) using a Gotaq PCR Master Mix (Promega, Madison, USA) on a CFX96 Real-Time System (Bio-Rad Laboratories, Hercules, USA). All reactions were performed in triplicate. The GAPDH was used as an endogenous control. Detailed primer information for each molecule was provided as follows:

P21 forward: 5′-ATGAAATTCACCCCCTTTCC-3′.

P21 reverse: 5′-CCCTAGGCTGTGCTCACTTC-3′.

MDM2 forward: 5′-ATCTTGGCCAGTATATTATG-3′.

MDM2 reverse: 5′-GTTCCTGTAGATCATGGTAT-3′.

PUMA forward: 5′-GACGACCTCAACGCACAGTA-3′.

PUMA reverse: 5′-AGGAGTCCCATGATGAGATTGT-3′.

BAX forward: 5′-GAGCTGCAGAGGATGATTGC-3′.

BAX reverse: 5′-GTTCTGATCAGTTCCGGCAC-3′.

GAPDH forward: 5′-GGTCGTATTGGGCGCCTGGTC-3′.

GAPDH reverse: 5′-GCCAGCATCGCCCCACTTGA-3′.

### Western blot analysis

Western blot analysis was conducted by standard protocols. Cells were cultured in dishes and treated with different treatments, and cell lysates were resolved on SDS-PAGE gels to transfer on to polyvinylidene difluoride membranes. The relevant antibodies were used to probe against specific primary antibodies against p21 (Cell Signaling Technology, MA, USA), MDM2 (Abcam, Cambridge, UK), p53, PUMA, BAX, PARP (Santa Cruz Bioechnology, CA, USA), and GAPDH (Beyotime, Shanghai, China), followed by HRP-conjugated secondary antibodies from Santa Cruz Biotechnology. Antigen-antibody complexes were detected by Bio-Rad Clarity™ western ECL substrate (Bio-Rad Laboratory, CA, USA).

### shRNA p53 knockout

Gastric cancer cells (AGS, MNK45) were transfected with lentiviral vectors expressing shRNA-p53 or scrambled sequence control shRNA (GenePharma, Suzhou, China) for 3 days and selected with puromycin (sigma, Shanghai, China) for 2 weeks.

### Apoptosis analysis by flow cytometry

Gastric cancer cells (1 × 10^5^ cells) were seeded in six-well cell culture cluster and treated with each regimen for 48 h. The cells were washed with 1× binding buffer and incubated with 5 μl of Annexin V/FITC (Annexin V-Alexa Fluor 488/PI Kit; Beijing 4A Biotech Co. Ltd. China) for 5 min in the dark at room temperature. The stained cells were analyzed with a ACEA NovoCyte™ flow cytometry (ACEA Biosciences Inc. China).

### Clonogenic assay

AGS and MNK45 were treated with DMSO and APG-115(20 nmol/L, 200 nm/L). Cells were irradiated with 0, 2, 4, 6, or 8Gy at dose rate of 1.8Gy/min, by use of X-ray irradiator RS2000 (RAD SOURCE, USA). The cells were incubated at 37 °C for 14 days after irradiation. Then cells were fixed and stained for 15 min in 70% and 0.5% crystal violet. Colonies were calculated by use of 50 viable cells. Plating efficiency was calculated by dividing the average number of cell colonies per well by the amount of cells plated. Survival fractions were calculated by normalization to the plating efficiency of appropriate control groups.

### Cell cycle analysis

After treatment with each regimen for 48 h, cells were harvested and fixed in 70% ethanol for at least 24 h. DNA was stained with propidium iodide solution (Beijing 4A Biotech Co. Ltd. China) according to the manufacturer’s protocol. DNA content was analyzed with an ACEA NovoCyte™ flow cytometry (ACEA Biosciences Inc. China).

### In vivo animal models

Four-week-old male BALB/c athymic nude mice were purchased from Beijing Vital River Laboratory Technology Co. Ltd. All animal experiments were performed under the guidance of Sun Yat-Sen University Committee for Use and Care of Laboratory Animals and approved by the animal experimentation ethics committee. MKN45 cells (5 × 10^6^) were combined with Matrigel (Corning, NY, USA) by 1:5 and then injected subcutaneously into the right infra-axillary of male BABL/c nude mice. When the tumors reached 100 mm^3^ in size, eight mice per group were subjected to treatment with radiation (delivered in five fractions of 2 Gy on days 1 to 5), APG-115 (delivered orally at 100 mg/kg once daily on days 1 to 10), or both. The control group was treated with vehicle control (1% Klucel LF/0.1% Tween 80). Tumor sizes and animal weights were recorded two times per week and tumor volumes were calculated as V (mm^3^) =1/2 × (length × width^2^).

### Immunohistochemical staining

Paraffin sections of xenograft tumor tissue were stained immunohistochemically using MDM2, p53, p21, PUMA and Ki67 antibody (1:500 dilution) and visualized using Leica microscope (Leica microsystem, Wetzlar, Germany).

### Statistical analysis

All statistical analyses were performed by Prism 5 (GraphPad Software). All statistical analyses were expressed as means ± SD (*p-value < 0.05). Experiments were conducted in duplicate or triplicate. Statistical analysis was analyzed by two-sided Student’s t test and ANOVA with Kruskal-Wallis test.

## Results

### APG-115 inhibited proliferation of gastric cancer cell lines

We evaluated p53, MDM2 protein levels in six untreated gastric cancer cell lines by western blot (Fig. 1b). Only AGS and MKN45 cell lines were p53 wild type, and they harbored MDM2 expression. To assess the inhibition effect of APG-115 in gastric cancer cell lines, we used CCK-8 method and found that APG-115 inhibited cell proliferation in six gastric cell lines in a concentration-dependent manner in 72-h. The IC_50_ of AGS and MKN45 were 18.9 ± 15.6 nM and 103.5 ± 18.3 nM respectively (Fig. 1c, d) and the IC_50_ of other gastric cancer cells were shown in Table 1. We found that four gastric cancer cell with p53 deletion and p53 mutation were not sensitive to APG-115 and the IC_50_ were more than 10 uM.

**Table 1:** The IC_50_ of APG-115 in six gastric cancer cell lines with different p53 status

Cell lines	p53 Status	IC_50_ for APG-115 (nM)
AGS	Wild-type	18.9 ± 15.6
MKN45	Wild-type	103.5 ± 18.3
BGC-823	deletion	> 10,000
SGC-7901	deletion	> 10,000
N87	Mutation	> 10,000
NUGC-3	Mutation	> 10,000

### APG-115 improved radiosensitization in human gastric cancer

**Fig. 2:**
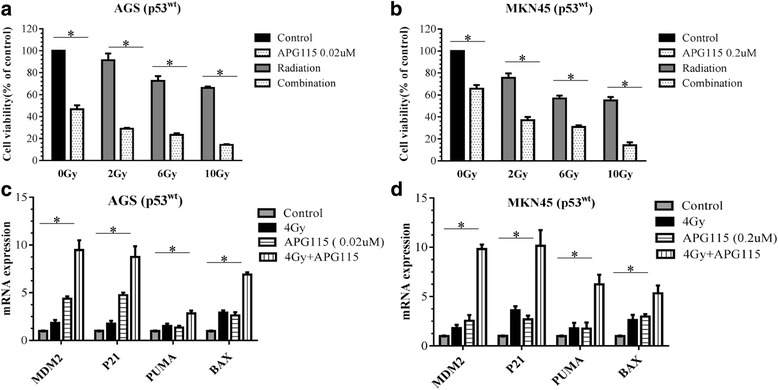
The cell proliferation assay demonstrating the cell viability of the AGS (**a**) and MKN45 (**b**) cell lines, expressed as a percentage of control group by the CCK-8 assay. AGS (**c**) and MKN45 (**d**) cell lines were treated with APG-115 (0.02 mol/L and 0.2 mol/L) and/or radiation (4Gy) for 0 to 48 h. Relative expression of MDM2, p21, PUMA and BAX mRNA compared with the control at 0 h was determined by RT-qPCR

To assess the effect of APG-115 on radiosensitivity in human gastric cancer, we evaluated cell viability after radiation with or without APG-115 treatment by the CCK-8 assay using AGS and MKN45 cell lines. The viability of both gastric cancer cell lines were decreased by APG-115 and radiation therapy in a radiation dose-dependent manner (Fig. 2a, b). Moreover, APG-115 increased the anti-proliferative effect of radiotherapy at different radiation dose. We also examined the effect of APG-115 alone or with radiation on mRNA expression of MDM2, p21, PUMA and BAX. Elevated expression of MDM2, p21, PUMA and BAX were observed in APG-115 group, radiation group and combination group. Compared with APG-115 group and radiation group, the mRNA expression of MDM2, p21, PUMA and BAX in combination group significantly increased (Fig. 2c, d).

### APG-115 radiosensitized gastric cancer cells and results in DNA damage in a p53-dependent manner

**Fig. 3:**
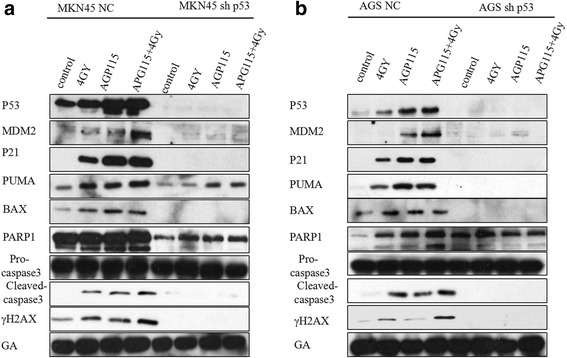
Western blot analysis shown the expression of proteins related to apoptosis and cell cycle arrest of MDM2-p53 signal pathway in AGS (**a**) and MKN45 (**b**) at 24 h

**Fig. 4:**
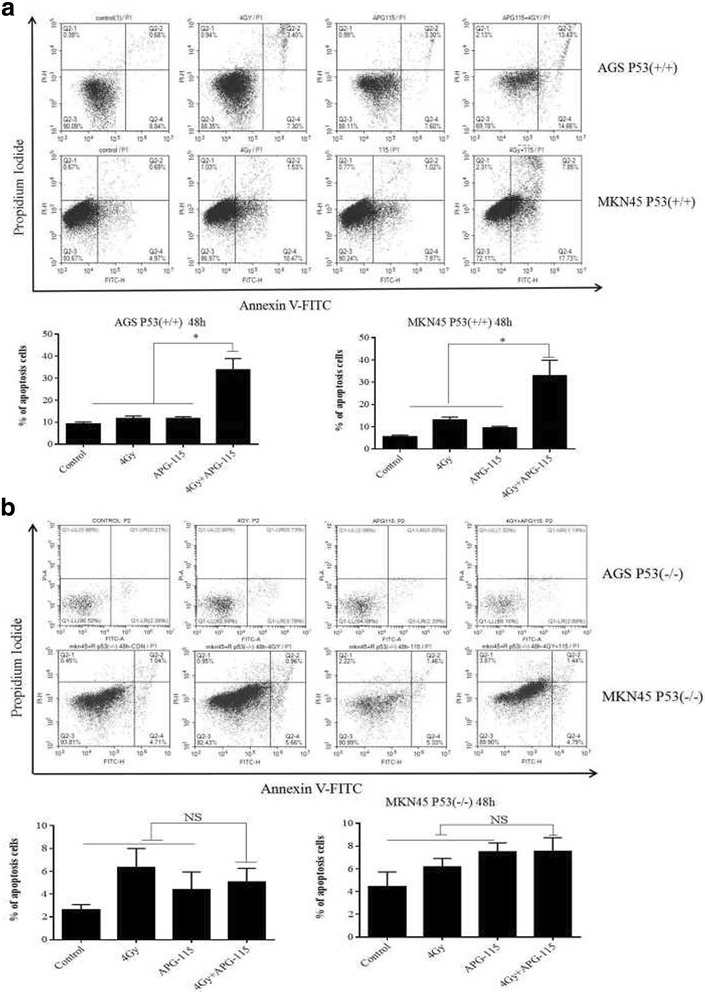
(**a**) Annexin/FITC analysis by flow cytometry showed the early and late apoptotic cells in AGS and MKN45 with p53 wild type at 48 h after different treatment. (**b**) In the p53 knockout AGS and MKN45 cell lines, there is no significant difference between combination group and single treatment group

To determine whether APG-115 sensitizes gastric cancer cells to ionizing radiation in a p53-dependent manner, we assessed the impact of MDM2 inhibition via APG-115 with radiation through western blot and flow cytometry analysis. Western blot analysis shown that the expressions of MDM2, p53, p21, PUMA, BAX, PAPR-1, Cleaved-caspase3, γH2AX in combination group were higher than other groups. In contrast, stable knockout of p53 in AGS and MKN45 cells effectively abrogated the expression of MDM2, p53, p21, PUMA, BAX, Cleaved-caspase3, γH2AX in different groups (Fig. 3). Next, we explored the role of apoptosis in radiosensitivity of APG-115. Apoptosis was analyzed via flow cytometry following treatment with APG-115 (20 nmol/L, 200 nm/L) alone or with/without radiation (4 Gy) in AGS and MKN45 with p53 wild type and p53 knockout. We found an increase apoptosis rate at 48 h for p53 wild type AGS and MKN45 among the three groups. The combination treatment of APG-115 and radiation resulted in a significant higher apoptosis rate compared with treatment with single therapy (Fig. 4a). The proportion of apoptotic cells in combination group was significantly higher than that in radiation and APG-115 group (AGS: 28.09 ± 1.4% (combination group) vs 10.7 ± 0.9% (radiation group), 10.8 ± 1.3% (APG-115 group), *p* < 0.05; MKN45: 25.58 ± 2.3% (combination group) vs. 12.0 ± 1.2% (radiation group), 8.99 ± 0.8% (APG-115 group), *p* < 0.05). The addition of APG-115 to radiation did not result in radiosensitization in p53 knockout AGS and MKN45 cell lines, AGS with p53 knockout: 4.51 ± 2.1% (radiation group), 2.93 ± 1.7% (APG-115 group) vs. 3.83 ± 0.8% (combination group), *p* > 0.05; MKN45 with p53 knockout: 6.62 ± 0.7% (radiation group), 6.79 ± 2.2% (APG-115 group) vs.6.23 ± 1.5% (combination group), *p* > 0.05 (Fig. 4b). These results suggest that APG-115 radiosensitizes gastric cancer cells and results in DNA damage in a p53-dependent manner.

### APG-115 significantly enhanced the radiosensitivity of AGS and MKN45 with wild type p53 and induced cells arrested at G0/G1 phase

**Fig. 5:**
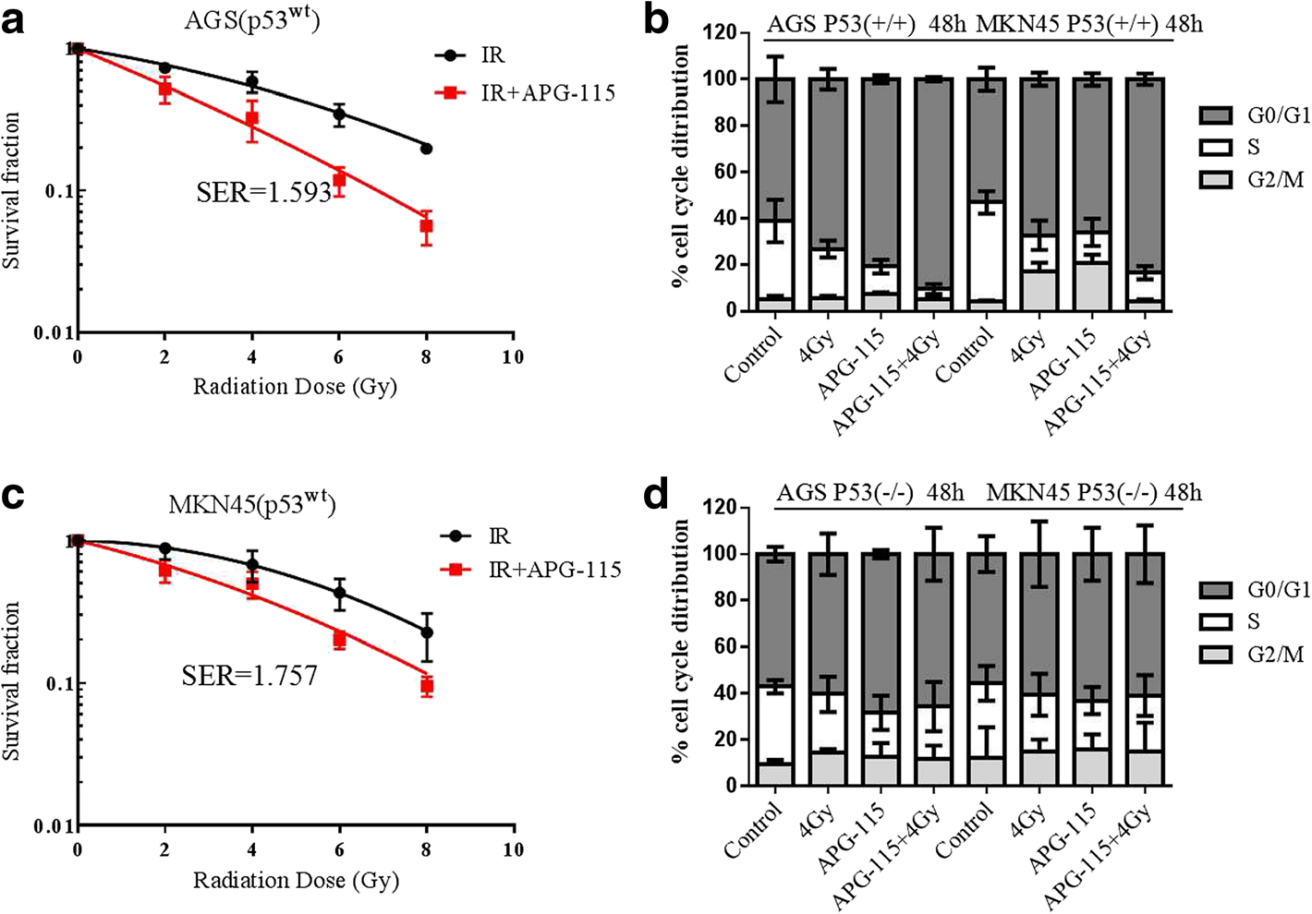
Clonogenic assays were performed to comfirm that APG-115 can enhance the radiosensivitity of AGS and MKN45 cells with wild type p53 (**a**, **b**). Cell cycle analysis showed that APG-115 enhanced radiation-inducing G0/G1 phase arrest in AGS and MKN45 cells at 48 h (**c**), but not in AGS and MKN45 cells with p53 knockout (**d**)

To determine the effect of APG-115 administration on radiosensitivity of AGS and MKN45, we used clonogenic assays to confirm it. We found that treatment of AGS and MKN45 cancer cells with different concentration of APG-115 (20 nmol/L, 200 nm/L) before radiation caused a remarkable curve shift in both cells compared with DMSO-treated cells. The sensitivity enhancement ratio (SER) were 1.593 and 1.757 respectively (Fig. 5a, b *p* < 0.05). To determine if APG-115 affects progression through the cell cycle, both AGS and MKN45 cells were treated with vehicle control, APG-115, radiation and APG-115 combined with radiation group. The percentage of cells in each stage of the cell cycle was then determined using flow cytometry. AGS cells treated with combination group had 90.4% of cells in G0/G1 phase, which was higher than other groups. The similarly results were also found in MKN45 cells, radiation vs. combination; AGS:73.3 ± 4.3% vs. 90.4 ± 1.0%, MKN45: 67.32 ± 2.8% vs. 83.4 ± 2.5%, *p* < 0.05 (Fig. 5c). There was no significant change in G0/G1 phase in AGS and MKN45 cancer cells with p53 knockout with different treatment (Fig. 5d).

### APG-115 suppressed tumor growth and enhanced anti-tumor effect by radiation in a xenograft mouse model

**Fig. 6:**
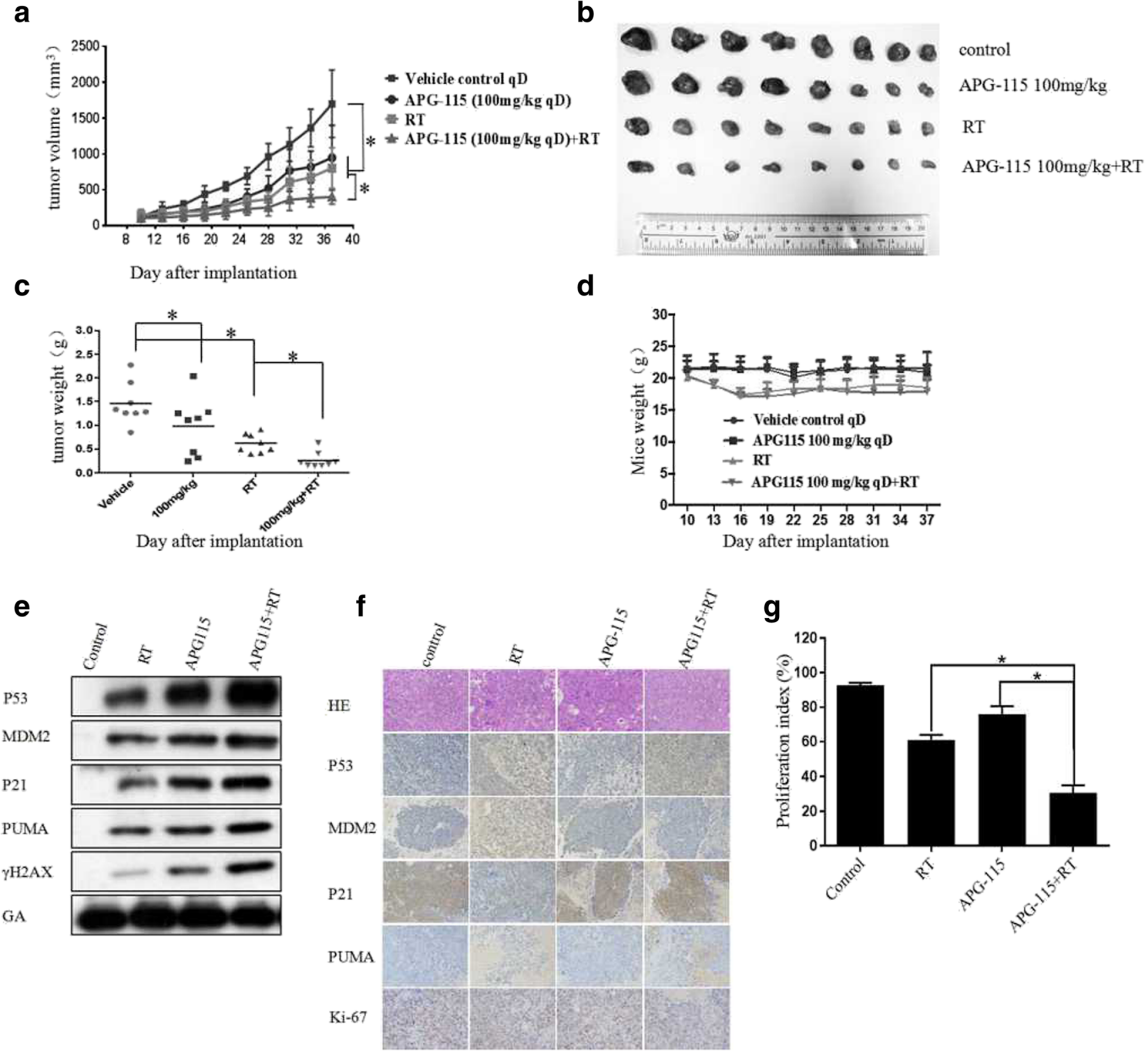
(**a**) shows changes in the tumor volume of the subcutaneous xenograft tumor in experimental groups. (**b**) The tumor volume in combination group was the smallest among the experimental groups. (**c**) The tumor weight in combination group was smaller than that in other groups. (**d**) There was no significant difference of weight in all experimental groups. (**e**) Western blot analysis shown the expression of proteins related to MDM-p53 signal pathway and DNA damage in xenograft tumor model (MKN45). (**f**) The expression of p53, MDM2, p21, PUMA and Ki67 in experimental tumors. (**g**) Quantification of the proliferation index in the tumor sections

To evaluate the anti-tumor activity by combination of radiation with APG-115 in vivo, nude mice bearing the xenograft was treated with vehicle control, APG-115, radiation and combination. APG-115 or radiation alone significantly suppressed the growth of gastric xenograft tumors (Fig. 6a, b). Meanwhile, APG-115 significantly enhanced anti-tumor effect in combination group (Fig. 6a, b). After 4 weeks of treatments, the tumor volume and tumor weight in the combination group were reduced by more than 50% compared with those in radiation group (Fig. 6a, b, c). There were no significant differences in the body weight between radiation and combination group (Fig. 6d). Consistent with the in vitro data, the protein expression in xenograft tumors treated with radiation and APG-115 were the same with those observed in vitro (Fig. 6e). Immunohistochemical staining showed that the percentage of Ki67 positive cells in combination group was significantly lower than that of radiation or APG-115 group (Fig. 6f, g).

## Discussion

Approximately 30% of gastric cancers have been found as p53 mutation or deletion [26]. MDM2 expression is positively regulated by p53 transactivation by feedback loop. Amplified MDM2 has been reported in > 10% of gastric cancers [27]. Under normal circumstances, p53 is controlled by MDM2 through feedback inhibition [28]. DNA damage resulted by ionizing radiation can activates the ataxia-telangiectasia mutated (ATM) kinase and p53 tumor suppressor protein. ATM phosphorylation by MDM2 is required for p53 stabilization and activation response to DNA damage. MDM2 attenuates p53 functions in cell cycle arrest, apoptosis, and response to DNA damage [29]. MDM2 activation may play an important role in preventing uncontrolled cell death caused by p53 in response to radiation [30]. An impaired p53 signal pathway plays a critical role in the carcinogenesis of GC. Restoration of p53 signal by MDM2-p53 inhibitor is a very attractive therapeutic strategy for the treatment of p53 wild type cancers. Ionizing radiation can activate p53 pathway, which leads to the expression of p21 and other downstream proteins, leading to cell cycle arrest and apoptosis [31–33]. Meanwhile, some studies with small-molecule inhibitors of MDM2 increased the expression of p53 and p21, inducing cell cycle arrest at G1/S and/or G2/M phase [34] and apoptosis [25, 35]. MDM2-p53 inhibitors can restore p53 activity in tumors harboring wild type p53 alleles and might also improve effect of radiation therapy. Previous studies have shown that restoring the balance between p53 and MDM2 can enhance radiosensitivity [36–39]. MDM2-p53 inhibitors are considered to be a type of valuable drugs in cancer therapeutics. It can activate p53 to enhanced radiosensitivity in different kind of cancers [23]. To our knowledge, there is no research concerning radiosensitivity of MDM2-p53 inhibitors in gastric cancer. In our study, we found that APG-115 significantly inhibited cell viability, induced cell cycle arrest at G1 phase and enhanced radiosensitivity of gastric cancer in vitro and in vivo. The mechanism of radiosensitivity by APG-115 not only depended on MDM2-p53 signal pathway, but might also include tumor microenvironment regulation [40]. Restoration of p53 by MDM2-p53 inhibitors may also raise concerns about toxicity to healthy tissues. Radiosensitive tissues, such as small-intestine, are especially susceptible to p53-induced apoptosis [41]. In previous studies, several MDM2-p53 inhibitors show little toxicity to animal models [35, 42]. Meanwhile, MDM2-p53 inhibitors combined with radiationtherapy is safe and tolerable [23, 24]. In our study, there is no obvious weight loss in combination group compared to each single group and no animal death in all groups. The remarkable antitumor capacity of APG-115 and radiation observed in vivo studies indicated that APG-115 may be a good radiosensitizer without obvious toxicity. In summary, our results demonstrated that APG-115 enhanced radiation-induced apoptosis and cell cycle arrest by MDM2-P53 signal pathway in gastric cancer in vitro and in vivo. APG-115 combined with radiation needs further clinical trial to confirm the anti-tumor effect in GC.

## Conclusions

Our results suggest that APG-115 combined with radiotherapy can enhance antitumor effect in MDM2-p53 signal pathway. APG-115 would be suitable as a radiosensitizer which need further clinical trial to confirm.

## Additional file

Additional file 1: The affinities to MDM2 of several MDM2-p53 compounds. (DOCX 16 kb)

## Abbreviations

ATM: Ataxia-telangiectasia mutated
CCK-8: Cell Counting Kit-8
GC: Gastric Cancer
MDM2: Murine double minute 2
RT-PCR: Real-time PCR
