# Supplementary material for: A novel small molecule inhibitor of MDM2-p53 (APG-115) enhances radiosensitivity of gastric adenocarcinoma
Source: J Exp Clin Cancer Res. 2018 May 2;37:97. doi: 10.1186/s13046-018-0765-8 (PMC5930807; doi:10.1186/s13046-018-0765-8)
Supplement: Supplementary file 1 — The affinities to MDM2 of several MDM2-p53 compounds. (DOCX 16 kb) [file 13046_2018_765_MOESM1_ESM.docx]

Table S1 The percentages of apoptosis in different treatment groups at 48h and 72h (p>0.05).

|  | | **The percentages of apoptosis at 48h** | **The percentages of apoptosis at 72h** |
| --- | --- | --- | --- |
| **AGS P53^(+/+)^** | APG-115 group | 10.8 ± 1.3% | 11.9± 3.6% |
|  | Radiation group | 10.7± 0.9% | 9.5±2.7% |
|  | Combination group | 28.09 ± 1.4% | 31.23 ± 3.6% |
| **MKN45 P53^(+/+)^** | APG-115 group | 8.99 ± 0.8% | 11.3± 2.5% |
|  | Radiation group | 12.0± 1.2% | 14.4±2.3% |
|  | Combination group | 25.58 ± 2.3% | 29.6 ± 1.4% |
